# Improving bereavement outcomes in Zimbabwe: results of a feasibility cluster trial of the 9-cell bereavement tool

**DOI:** 10.1186/s40814-023-01313-2

**Published:** 2023-07-21

**Authors:** Barbara Mutedzi, Kennedy Nkhoma, Lisa Langhaug, Jennifer Hunt, Richard Harding

**Affiliations:** 1 Island Hospice and Healthcare, 6 Natal Road, Belgravia, Harare, Zimbabwe; 2 Florence Nightingale Faculty of Nursing, Midwifery and Palliative Care, Cicely Saunders Institute of Palliative Care, Policy and Rehabilitation, King’s College London, Bessemer Road, London, SE5 9PJ UK; 3 Zvitambo Maternal and Child Health Research Institute, 16 McLaughlin Road, Harare, Zimbabwe; 4 Harare, Zimbabwe

**Keywords:** Grief, Bereavement, Nine-cell tool, Community-based, Feasibility cluster trial, Palliative care, Africa, Zimbabwe, Mental health

## Abstract

**Context:**

Despite high mortality rates from both communicable and non-communicable diseases, bereavement is under-researched in African countries. The 9-cell bereavement tool was designed to assist individuals to reflect on their feelings about bereavement and identify resources in families and communities to manage bereavement. This study aimed to determine the feasibility of implementing the 9-cell bereavement tool and recruitment to experimental evaluation.

**Methods:**

A feasibility cluster randomized trial with embedded qualitative interviews was conducted in two comparable neighbourhoods in Chitungwiza, Zimbabwe. Community leaders identified potential community lay bereavement supporters (interventionists). Each community lay bereavement supporter recruited two to three recently bereaved community members (trial participants). Following baseline data collection, the communities were randomly allocated to intervention or wait-list control. Self-administered questionnaires were completed at T0 (month 0), T1 (3 months) and T2 (6 months). Grief, mental health and social support were assessed. Focus group discussions with selected interventionists described training impact and intervention processes. Quantitative and qualitative analyses were performed.

**Results:**

Implementation of the nine-cell bereavement tool and recruitment to experimental evaluation were successful. Implementation of the tool and the recruitment of study participants were conducted within the intended timeframe of 3 weeks. In line with the suggested sample size, the study was able to recruit and retain at least 75% of the trial participants for the total duration of the study.

**Conclusion:**

The feasibility cluster trial was successfully implemented and assessed. Through the published protocol, the literature review and the results of this study, it has been noted that there is an urgent need to carry out a full trial in this subject matter, not only as a contribution to the currently sparse literature in this regard, but for the enormous potential public health benefit in supporting and saving lives in many more under-resourced and under-supported countries.

**Trial registration:**

Protocol registration: http://www.isrctn.com/ISRCTN16484746.

Protocol publication: https://pilotfeasibilitystudies.biomedcentral.com/articles/10.1186/s40814-019-0450-5

**Supplementary Information:**

The online version contains supplementary material available at 10.1186/s40814-023-01313-2.

## Key messages regarding feasibility

- Uncertainties that existed regarding the feasibility: Uncertainties included whether the study would be able to recruit and retain participants for the duration of the study pegged at 10 months with 3 different data collection time points. The uncertainty with the recruitment process was that it was not a linear process as it included several stages that included community entry, permission from local authorities and support from community members and interventionists. With retention, the uncertainty revolved around the need to retain at least 75% of the participants throughout the duration and stages of the study, whilst keeping in mind any circumstances that would hinder progress, communication with participants and any national or community challenges that may present during the study. Given the length of the study, additional uncertainties involved the possibility of contamination between the control and the intervention group and completeness of data that may be caused by possible participant fatigue in contributing to a study over 10 months. All these would affect the ability to collect data on participants’ views of the intervention toward the end of the study, which would, in turn, reduce the possibility of estimating the potential effect size as well as presenting contributory information to determine whether a full trial would be warranted.
- Key feasibility findings: In line with the aim and objectives of the study, all the processes of the randomized cluster trial were possible. It was feasible to recruit and retain 75% of the participants; there were no reported incidences of contamination, data was successfully collected at all time points and analysed data shows that the nine-cell bereavement tool was effective in allowing interventionists to share and learn from their own grieving process. This was the first time this locally and contextually developed 9-cell bereavement tool was tested and successfully implemented in Zimbabwe. The feasibility of recruiting, retaining and delivering the intervention indicates that a full trial is warranted.
- The implications of the feasibility findings for the design of the main study: Collectively, our recruitment and delivery processes work and can be used for a large main trial. Additionally, a full trial will contribute to the currently sparse literature on bereavement and bereavement interventions as an essential and core component of palliative care in sub-Saharan Africa and will have an enormous, potential public health benefit in supporting and saving lives in many more under-resourced and under-supported countries.

## Introduction

Despite high mortality rates due to communicable diseases such as HIV, tuberculosis and malaria and non-communicable diseases such as cancer, heart disease, suicide and sudden deaths, bereavement is an under-researched field in African countries [1, 2]. The WHO Global Palliative Care Atlas [3], the Lancet Commission on Pain and Palliative Care [4] and Universal Health Coverage [5] all identify a critical gap between the need for and provision of palliative care, including bereavement care.

Bereavement interventions are rarely described within African palliative care intervention studies [6–8]. This is urgently needed, given projections that by 2060, 48 million people will die with serious health-related suffering, 83% of these in low- and middle-income coutries [9]. Serious health-related suffering will increase in all regions, with the largest proportional rise in low-income countries (155% increase between 2016 and 2060). Each of these deaths with suffering will significantly impact the family and community of the decedent.

Bereavement is the process during which grief is experienced over time [10]. Bereaved individuals who have not been through the process of grief have an increased risk of mortality [11, 12], deterioration of physical health [11], reduced cognitive functioning and an increase in mental health challenges and associated illnesses [13]. These outcomes negatively impact the socio-economic status of individuals whilst generating high costs in already fragile economies of low-income countries [14, 15]. Bereavement support is therefore an essential and core component of palliative and end-of-life care [16, 17].

Community-based interventions using already existing structures, for example, community lay health workers embedded in the local health system, are more effective and widely accepted within low-resourced countries such as Zimbabwe, whose socio-economic structures have vastly deteriorated [18]. Previous studies have shown that community lay health workers trained to act as an extension of the central health centres are effective in health delivery as they increase coverage and access [19]. Caregivers from the communities that local health services serve can offer in-depth knowledge of local cultural preferences and practices for effective delivery and uptake of healthcare services [20–22].

This study investigates the ‘9-cell bereavement intervention’ developed in Zimbabwe. The 9-cell was designed to assist individuals to reflect on their feelings about bereavement and identify resources in families and communities to manage bereavement. This process is intended to increase the lay supporter’s understanding of the experience of grief and to identify ways of increasing support to the bereaved. The tool explores the communication that the bereaved currently receive, discussing and linking their own grief and bereavement experiences with the support they need at different stages of the grieving process [23].

This person-centred approach is designed to provide context-based, culturally appropriate and individually tailored support. Developed in Zimbabwe, this approach has been delivered in emergency contexts and within bereaved communities in Tanzania and India, with process data suggesting an increase in awareness of the concepts behind grief and the bereavement process, but not fully evaluated [24]. Evidence for culturally appropriate person-centred care in the context of serious illness and bereavement is scarce [25], especially in sub-Saharan Africa [26–28].

This study aimed to determine the feasibility of implementing the nine-cell bereavement tool and recruitment to experimental evaluation. The feasibility questions and criteria were as follows: (i) Will the process of a randomized cluster trial be possible? (ii) Will we be able to recruit at least 75% of the suggested sample size within 3 weeks? (iii) Will we be able to retain at least 75% of the trial participants in the 9 months of the study? (iv) Can we deliver the 9-cell bereavement intervention? (v) Is the intervention delivered as intended and does process data suggest the planned effect is likely?

The study/trial objectives were (i) to determine the feasibility of conducting a randomized cluster trial in terms of recruitment and retention, (ii) to assess the feasibility of implementing the nine-cell bereavement tool, (iii) to determine whether there would be contamination between the clusters, (iv) to assess the acceptability and completeness of measures and data, (v) to identify trial participants’ views and experience of the intervention and its mechanisms of action, (vi) to estimate potential effect size and (vii) to determine whether a full trial is warranted.

## Methods

### Setting

This feasibility trial was carried out in Chitungwiza: a high-density dormitory town, populated at 456,000 at the time of the study, and situated approximately 30 km from the capital city of Harare, Zimbabwe. The selected study sites were two comparable suburbs in Chitungwiza, but they were 8 km apart to reduce the risk of contamination.

As per the study protocol [29], the potential vulnerability of bereaved individuals and the scarcity of resources within sub-Saharan Africa were essential to establish feasibility prior to investment and participant involvement in a full clinical trial. In addition, cluster randomization is required in a full trial as the intervention is delivered at the community level. As a result, this study was conducted using a cluster randomized control trial design.

A cluster approach was used to assess the effectiveness of the nine-cell bereavement tool. Cluster RCTs are described as experiments in which intact social clusters are randomly allocated to the intervention groups [30].

A local organization, Island Hospice and Healthcare Zimbabwe (IHH), collaborated on the study given their longstanding involvement in Chitungwiza and connections to the local community groups, churches and local government structures. Lay community health workers, were recruited in partnership with already existing community leaders that Island Hospice and Healthcare Zimbabwe work within other programmes in Chitungwiza.

### Procedures

#### Recruitment

Recruitment was planned for 3 weeks and through three stages.

##### Week One

Stage One Recruitment: Community leaders, who collectively represented the two sites in Chitungwiza, were identified through Island Hospice and Healthcare, Zimbabwe. A meeting was held to share and explain the rationale, goals and intended procedures of the study. Permissions to conduct the study in their respective communities were sought and granted. In addition, a request for their assistance in the first stage of recruitment was sought and accepted.

Stage Two Recruitment: Once approval to carry out the study, and to assist in the first stage of the recruitment process, was accepted, the community leaders were tasked to recruit 25 lay community health workers from each of the two sites. Recruitment involved asking the recruited lay community health workers to attend a meeting where information regarding the study would be shared with them. The inclusion criteria meant that the community leaders were advised to invite community lay health workers who they knew to have suffered loss in the past 6 to 18 months to attend the meeting. These became potential interventionists.

As per the protocol [29], interventionists had to have had recent bereavement experiences. Previous research indicated that interventions were more effective when administered closer (though not too soon) to the time of death of the loved one [29, 31–33]. Additionally, research suggests that 6 to 18 months is a suitable period for administering bereavement interventions inclusive of their follow-up [33].

##### Week Two

The potential interventionists met in their respective communities and on different days and times, as informed at recruitment. Information regarding the study was shared and explained, with an invitation to take part in the study. Written informed consent was given to represent understanding and a willingness to participate in the study.

Once written informed consent was given, interventionists were provided with a short, self-administered questionnaire that assessed their socio-demographic background and bereavement history. Trained research assistants provided instruction to the interventionists and were available to assist with clarification of any questions where required.

Each cluster or site was notified that they would receive the intervention, either earlier on in the course of the study or toward the end of the study. Neither cluster knew when the other was going to receive the intervention, and if and when the other was participating in the study. During the explanation of the study to either clusters, no mention of the other cluster was made.

Stage Three Recruitment: On completion of the self-administered questionnaire, the interventionists from each community were tasked with identifying two to three people according to the following criteria: (a) at least 18 years old, (b) resident within their neighbourhoods, (c) someone with whom they interacted with on a daily basis, (d) someone whom they knew to have been bereaved in the past six months, (e) someone who would have the ability to either verbally consent or be able to provide written consent and (f) someone who could be expected to attend and participate in the study.

At the same time, a focus group discussion with a sub-sample of the interventionists was conducted to assess the feasibility of them identifying potential trial participants and inviting them to meet with the researchers to learn more about the study. Once completed, each interventionist was provided with three invitation letters to give to the two to three people they would have identified under this inclusion criteria.

The invitation letters had the name of the potential trial participant, the name of the interventionist who gave them the invitation, and the venue, date and time for them to attend the meeting to learn more about the study. Each site had different dates and locations for the meetings.

##### Week Three

Potential participants who met all of the inclusion criteria attended a meeting on the dates, times and location listed on the invitation letters they were given. These letters served firstly, as ‘entry’ into the study centre. Secondly, they acted as an extra measure to curb any walk-ins from the rest of the community. Thirdly, they confirmed that all those who had the invitation letters fit the inclusion criteria to the study. Once everyone had arrived and their legitimacy was confirmed, the meeting started and information regarding the study was shared. Participants were invited to take part in the study; those who agreed provided written informed consent. These were now the study trial participants.

#### Sample size

This study targeted a sample of 100; two participants for each of the 50 interventionists, 25 interventionists from each site. Past research has recommended sample sizes of 24 and 50 [34–37]. The reason that each interventionist was asked to recruit two to three participants, as opposed to just two, which would add up to the targeted 50, was to allow for the possibility of some interventionists recruiting less than the minimum number of two; and in preparation of any recruitment challenges for some of the interventionists, and for any problems with retention drops that could be experienced over the course of the whole study.

Data from trial participants was collected at three time points: (i) baseline data (T0), (ii) midline data (T1) after 3 months and (iii) endline data (T2), after an additional 3 months. Data was collected on different days and times at each site and in their respective communities. Following the collection of baseline data (T0), the two study sites were randomized by a statistician (independent of the study) at Kings College London to intervention or wait-list control. The wait-listed community received the intervention at the end of the data collection.

#### Questionnaires used at baseline, midline and endline

Trial participants completed the self-administered questionnaires at baseline (T0), midline (T1) and endline (T2) and included the following primary assessment tools: (1) the Shona Symptom Questionnaire (SSQ) [38] a screening tool for mental health, (2) the Medical Outcomes Study—Social Support Survey (MOS-SSS) [39] which measures social support and the (3) Texas Revised Inventory of Grief (TRIG) [40, 41]which measures intensity of a person’s grief. Each questionnaire included a section on sociodemographics and bereavement history.

Included at the beginning of each data collection period, was a small and separate contamination questionnaire that asked if the participant had visited and or had contact with anyone who lived in the other cluster.

### The intervention

Description: The 9-cell bereavement tool’s structure (i) draws on participants’ existing knowledge, models an open-minded, non-judgmental approach; recognizes the diversity of grief within individuals, genders, families, cultures and faiths; and encourages participants to listen to others whilst breaking down previously held beliefs about how grief can be expressed. It draws on Stroebe and Schut’s oscillation model (1998) [42], which focuses on people’s oscillation between the process of grief in itself, together with re-engaging with a life transformed by the loss [43]. The intervention uses nine ‘cells’ to help an individual identify (i) personal feelings in relation to their bereavement; (ii) judgmental attitudes, religious tenets and lack of understanding and (iii) effects of family and community support.

**Fig. 1:**
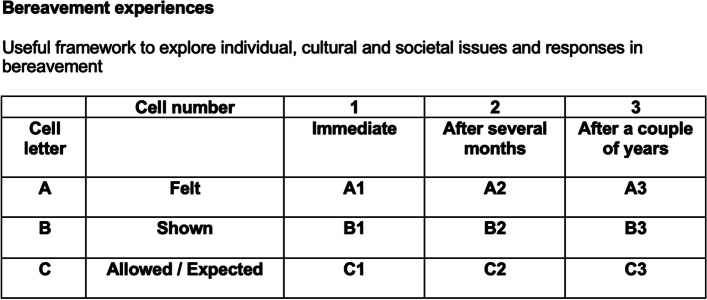
Nine-cell table example

Structure: During the intervention in both sites, a nine-cell table (see example in Fig. 1) was constructed. The horizontal line represented three time points after the loss of a loved one, that is (i) the immediate, (ii) a little whilst later and (iii) a long time after the event. The vertical divisions examine (i) the individual’s feelings, (ii) how these are outwardly expressed and (iii) what is culturally permissible.

Process: Discussions comparing the different cells in the nine-cell table after they were filled in, act as and are the intervention. Evidence-based alternative points are offered during the discussion for consideration. These points helped participants realize the gap between what they feel and what is permissible, and then they all worked together to develop a personal bereavement approach.

Discussions were allowed to take place in either direction, first examining feelings along a timeline, or exploring how expressions differ between immediate feelings, what is outwardly expressed and what is permissible. As with the intervention process, the facilitator merely asked for thoughts or personal experiences to be identified in each cell, probing for further details, differences between experiences in the group and, in particular, contrasting thoughts relating to rituals, religious teachings and individual interpretations of these, whilst offering optional ideas for reflection.

#### Post-intervention implementation

Focus group discussions (FGDs) were held with interventionists from the interventionist, site 1. The purpose of the focus group discussions were to examine the interventionists’ experience and use of the training material, implementation and processes within the intervention. Discussions were captured through detailed notes from the note-taker and translated from the vernacular (Shona) to English.

A focus group discussion (FGD) was held during pre-midline data collection (T1) with trial participants. This would have given about 3 months for interventionists to put into use the lessons learnt from the intervention.

#### Analysis

Trained researchers as per the published protocol [29] conducted the analysis. Quantitative analysis was conducted by KN (PhD, male statistician, research advisor), supported by RH (PhD, male research director, research director). Qualitative analysis was conducted by BM (MSc, female medical anthropologist, principal investigator) and supported by LFL (PhD, female social scientist, research consultant). Analysis was conducted in line with the study objectives, with feasibility questions and criteria included in the reporting.

Feasibility questions and criteria were as follows: (i) Will the process of a randomized cluster trial be possible? (ii) Will we be able to recruit at least 75% of the suggested sample size within 3 weeks? (iii) Will we be able to retain at least 75% of the trial participants in the 9 months of the study?; (iv) Can we deliver the 9-cell bereavement intervention? (v) Is the intervention delivered as intended and does process data suggest the planned effect is likely?

Feasibility questions and criteria (i), (ii) and (ii) are addressed in objective (i).

Feasibility questions and criteria (iv) are addressed within objective (ii).

Feasibility questions and criteria (v) are addressed within objectives (iii) to (vi).

The objectives were as follows:

i) To determine the feasibility of conducting a randomized cluster trial in terms of recruitment and retention
  Numbers of both interventionists and trial participants (i) recruited and (ii) participating in the trial were recorded at baseline (T0), midline (T1) and endline (T2). At the end of each data collection phase, researchers analysed their flow of work to assess what worked and what needed adjustment in allowing a conducive environment for optimum data collection from the participants. This included debriefs that the researchers conducted at the end of each data collection point. Where any process may have affected the resultant figures, these were documented in short reports to be used as reference points for the end of trial report.
ii) To assess the feasibility of implementing the 9-cell bereavement tool
  This was established in part through qualitative observation of the discussions that emerged during intervention implementation and through a discussion with the facilitators themselves post-study to assess (a) their experience in implementing the intervention and (b) their assessment of the experience of the interventionists as they participated in the intervention and (c) to highlight what made the process feasible. Framework analysis was used to analyse the qualitative data collected from the discussion. Framework analysis allows an in-depth analysis of data ‘simultaneously maintains an effective and transparent audit trail’ [44].
iii) To determine whether there would be contamination between the clusters
  Contamination questions were provided to both the intervention and the control group to assess whether any of the participants had engaged, visited or spoken to either party in between the data collection dates. Questions were centred on whether they had visited the other community and/or been in contact with a participant from that group. Responses were manually assessed to see if any had contact and had been ‘contaminated’.
iv) To assess the acceptability and completeness of measures and data
  Questionnaire data from quantitative data collected at baseline (T0), midline (T1) and endline (T2) were manually entered into a Microsoft Excel spreadsheet. These were then imported into the Stata V15 software for analysis [45]. All participants’ data were analysed according to the community in which they were recruited and randomized.
  We tabulated all the variables for each measure used from baseline, midline and endline and each variable with missing data was recorded with the reasons provided. We manually summed up the number of participants who completed measures at baseline, midline and endline. This process allowed for any challenges that data entry may pose in a larger trial.
v) To identify trial participants’ views and experience of the intervention and its mechanisms of action
  Two focus group discussions, 45 min long each, were held with the trial participants at the end of the trial. The first focus group discussion had 13 participants and the second had nine participants. All qualitative data were manually analysed using framework analysis within Microsoft Excel. Framework analysis with emerging themes around specific questions was used to analyse trial participants’ responses [44].
vi) To estimate the potential effect size
  We calculated the baseline and final scores for outcome measures (SSQ, TRIG and MOS-SSS) and summarized within-group changes for each outcome measures and for differences between communities at the final time point.
  We performed longitudinal analysis using multilevel modelling for repeated measures with generalized linear latent and mixed models (GLAMM) which accounts for correlated or clustered data over time in analysing categorical data. Each outcome was divided into quartiles, as GLAMM operates more successfully with fewer categories of the dependent variable, and was adjusted by baseline score. This enabled a comparison of the effect of the intervention on all dependent variables.
vii) To determine whether a full trial is warranted
  The combined results from the above analysis would warrant whether a full trial was possible, with the ability of conducting a full RCT in terms of recruitment and retention, successful implementation of the intervention, little or absent contamination between the clusters, acceptability and completeness and measures and data, identification of trial participant’s views and experience of the intervention and its mechanisms of action and the ability to estimate potential effect size.

#### Uncertainties that existed regarding the feasibility

Uncertainties were centred on the objectives and aim of the study. These included whether the study would be able to recruit and retain participants for the duration of the study pegged at 10 months with three different data collection time points per group. The recruitment process was staggered with several stages that included community entry, permission from local authorities and support from community members and interventionists. With retention, the uncertainty revolved around the need to retain at least 75% of the participants throughout the duration and stages of the study, whilst keeping in mind any circumstances that may hinder progress, communication with participants and any national or community challenges that may present during the study.

Taken from our protocol, there is a paucity of guidance on setting progression criteria for feasibility and pilot trials [46]. Therefore, we drew on the MRC guidance that such criteria should be judged in light of all study findings and used to refine the study design. Our recruitment and retention criterion of 75% was set in light of published feasibility trial criteria and reflects the nature of our population (i.e. they are community-dwelling bereaved individuals without any known serious health conditions and so we anticipate high retention) [29].

Given the length of the study, additional uncertainties involved the possibility of contamination between the control and the intervention group and completeness of data that may be caused by possible participant fatigue in contributing to a study over 10 months. All these would affect the ability to collect data on participants’ views of the intervention toward the end of the study, which would in turn, reduce the possibility of estimating potential effect size as well as presenting contributory information to determine whether a full trial would be warranted. Different measures were addressed to reduce the occurrences of these uncertainties. These are included at respective sections in the results section.

### Ethical approvals

Ethical approvals were obtained from the Zimbabwe National Medical Research Council of Zimbabwe (MRCZ/A/2230) and from King’s College London (HR-17/18–5415). In addition, the study sought clearance from the local community police and researchers carried all clearance documents with them throughout the study.

## Results

Results are reported with each corresponding objective. Embedded in the results that follow, are results on feasibility criteria.

**Objective 1: Determining the feasibility of conducting a randomized cluster trial in terms of recruitment over a 3-week period and retention of trial participants throughout the trial period**

Feasibility question (i) asked: Will the process of a randomized cluster trial be possible?

We found that it was feasible to conduct a randomized cluster trial.

Feasibility questions (ii) and (iii) asked: Will we be able to recruit at least 75% of the suggested sample size within 3 weeks, and will we be able to retain at least 75% of the trial participants in the nine months of the study? Answers to both questions were positive, as illustrated in the following section.

### Recruitment of interventionists

The study was able to recruit the suggested sample size within 3 weeks. Following the suggested trial recruitment criteria as indicated in the protocol [29], we managed to communicate and sensitize community leaders within 1 week; interventionists were recruited within 1 week, and the trial participants were successfully recruited within another week.

Feasibility study design sample size recommendations are for 24–50 per group. The intended minimum numbers to be recruited by the community leaders were, therefore, set at 50 interventionists for both sites combined. Recruitment by the interventionists was set to be a minimum of 100 trial participants for both sites. The interventionists were asked to recruit 2–3 participants, to allow for any challenges in retention and for possible attrition from the study.

The actual numbers recruited in a total of both communities were interventionists: 56, which is 12% more than the minimum suggested, and trial participants: 143, which is 43% more than the suggested minimum (see Tables 1 and 2).

Table 1 indicates both sample size, suggested recruitment figures and actual recruitment of interventionists.

**Table 1:** Interventionist recruitment rates

Interventionists	Target recruitment (*n*)	Actual recruited (*n*)	Percentage (%) of target actually recruited
Community 1	25	25	25 = 100%
Community 2	25	31	31 = 112%
Total	50	56	56 = 112%

### Recruitment of trial participants

Table 2 below indicates both sample size, suggested recruitment figures and actual recruitment of trial participants. As per recommended sample size [34, 35, 47, 48], a target of 100 trial participants was set, meaning 50 for each site.

**Table 2:** Trial participant recruitment rates

Trial participants	Target recruitment (*n*)	Actual recruited (*n*)	Percentage (%) of target actually recruited
Community 1	50–75	57	57 = 114%
Community 2	50–75	86	86 = 172%
Total	100–150	143	143 = 143%

We recruited a total of 143 trial participants (T0): 57 from the intervention group and 86 from the control group. We had 43 more participants (see Table 2).

### Retention rates of interventionists

The minimum retention rate was set to 75% from baseline to endline. Results show that the study had 100 + % retention rates. See Table 3.

**Table 3:** Retention rates of interventionists

	Actual recruited (*n*)	Actual retained at the midline (*n*)	Actual retained at the endline (*n*)	Actual (%) retained
Community 1 interventionists	25	25	25	100 + %
Community 2 interventionists	31	31	31	100 + %
**Total**	**56**	**56**	**56**	**100%**

### Retention rates of trial participants

At midline (T1), we retained 52 trial participants from the intervention group and 54 from the control group. At endline (T2), we retained 52 from the intervention group and 46 from the control group.

Despite the substantial loss to follow-up for the control group, that is 40 people between baseline and endline, our retention targets were not negatively affected due to the high recruitment rates (+ 43%). Actual figures from the study show that overall retention rates were 74% at 3 months (midline) and 69% at 6 months (endline) (see Table 4).

**Table 4:** Retention rates of trial participants

	Actual recruited	Actual retained at the midline	Actual retained at the endline	Actual (%) retained
Community 1 trial participants	57	52	52	52/57 = 91% base to the midline 52/52 = 100% mid to the endline 52/57 = 91% base to the endline
Community 2 trial participants	86	54	46	54/86 = 62% base to the midline 46/54 = 85% mid to the endline 46/86 = 53% base to the endline
**Total**	**143**	**106**	**98**	**106/143 = 74% base to the midline** **98/106 = 92% mid to the endline** **98/143 = 69% base to the endline**

Differential attrition between the intervention and control group is further explained under objective (iv) that speaks to the acceptability and completeness of measurement of data between baseline and midline, and midline and endline.

**Fig. 2:**
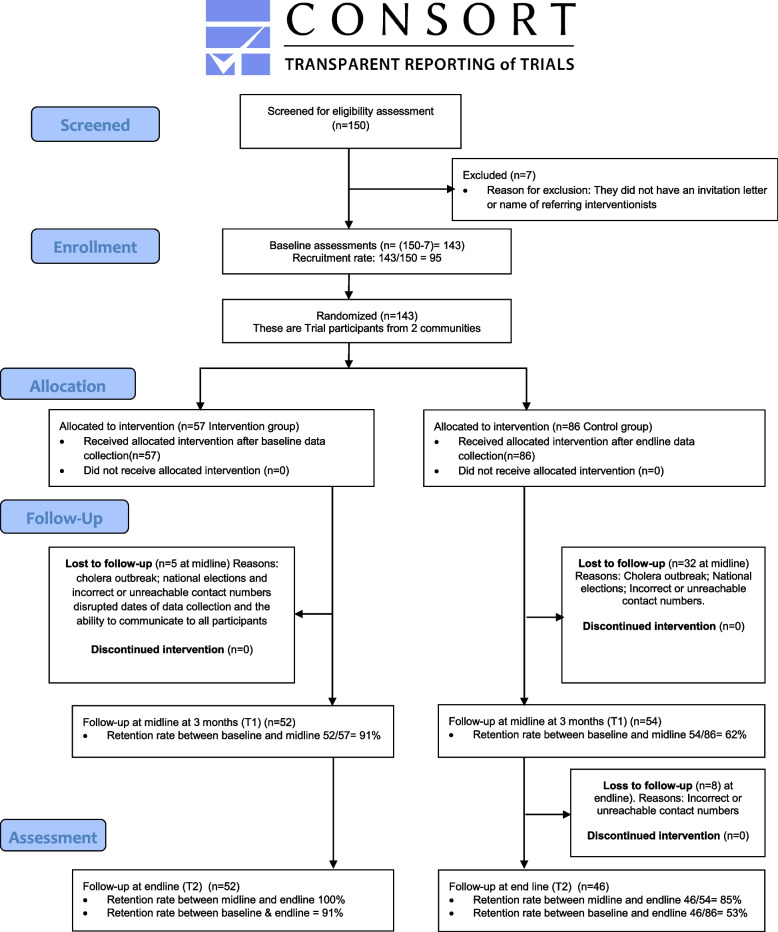
The CONSORT flow chart attached visually summarizes and illustrates the full recruitment and retention process and figures

Factors that impacted retention rates are illustrated in Fig. 2 and in the sections that follow.

**Objective 2: Feasibility of implementing the 9-cell bereavement tool**

Feasibility question (iv) asked: Can we deliver the 9-cell bereavement intervention? The results were positive as indicated in the following section.

### Intervention delivery, adherence and impact

The two facilitators ascertained that the intervention was delivered correctly. This was established in part through qualitative observation of the discussions that emerged during intervention implementation and through a discussion with the facilitators themselves post-study.

They [interventionists were] fully engaged… they worked hard…we had brilliant results. It was an extremely positive experience: (Intervention Facilitator One)

As they went through the nine cells, the facilitators found interventionists processing unresolved past grief and reflecting on the cultural expectations around discussing the loss of a loved one. Interventionists shared their own stories of grief, and their comments indicated that they were reflecting on the processes:

They [interventionists] were every engaged from the outset. They were able to understand what the intervention was about. They were able to share about their own grief and reflect on their emotions: (Intervention Facilitator One)

Just creating a supportive and safe atmosphere to express their grief: that facilitated the entire process right until the end. They [interventionists] were free and were supported throughout the process: (Intervention Facilitator ID Number Two)

One of the observations made by the facilitators of the nine-cell grief and bereavement tool was that they recognized that a once off single session may not always yield 100% positive change for all interventionists who take part in the nine-cell bereavement intervention as it may take more than the 1-day session for interventionists to reshape people’s manner of fully expressing their grief. Some participants may need more time to absorb the value of this new approach.

Objectives 3–7 address and indicate feasibility question (v) that asks: Is the intervention delivered as intended and does process data suggest the planned effect is likely? Results were positive to this effect and illustrated in the data that follows.

**Objective 3: Determining whether there would be contamination between the clusters**

Contamination screening questions completed by trial participants in both communities found that no contamination was reported.

**Objective 4: To assess the acceptability and completeness of measures and data**

Results in this section are not statistically significant because the study was not powered to detect significance. However, we have included them, as they can provide parameter estimates for powering a full trial.

Baseline data were collected from all 143 participants before the communities were randomly allocated to either receive the nine-cell intervention (*n* = 57, the intervention group) or be a part of the control group (*n* = 86, the control group) (see Table 1).

At baseline, participants’ demographic characteristics in the two communities were not similar. For example, there were many more participants aged 18–25 years in the control group compared to the intervention group (*n* = 17; 20% vs *n* = 3; 5.26%), many more participants were formally employed in the control arm compared with the intervention arm (*n* = 20; 23.26% vs *n* = 6; 10.53%) and many more participants from the control group had secondary education (*n* = 62; 72.09% vs 28; 49.12%) (see Tables 5, 6, and 7 on demographics and outcome scores per community).

**Table 5:** Demographics of the comparison of the participants from the two communities

Variables	Intervention (Seke) (*n* = 57)	Control (St. Mary’s) (*n* = 86)
Age in years^a^		
18–25	3 (5.26)	17 (20)
26–35	11 (19.30)	19 (22.35)
36–45	16 (28.07)	25 (29.41)
46–55	13 (22.81)	14 (16.47)
46/max	14 (24.56)	10 (11.76)
Male/female gender	11 (19.30)/46 (80.70)	13 (15.12)/73 (84.88)
Black race/other races	56 (98.25)/1 (1.75)	85 (98.84)/1 (1.16)
Importance of religious beliefs		
Not important at all	1 (1.75)	0
Not very important	1 (1.75)	1 (1.16)
Fairly important	2 (3.51)	1 (1.16)
Very important	53 (92.98)	83 (96.51)
Do not know	0	1 (1.16)
Religion		
Catholic	7 (12.28)	9 (10.47)
Presbyterian	15 (26.32)	17 (19.77)
Apostolic	10 (17.54)	19 (22.09)
Pentecostal	18 (31.58)	26 (30.23)
Others	7 (12.28)	15 (17.44)
Employment		
Not employed	18 (31.58)	29 (33.72)
Formally Employed	6 (10.53)	20 (23.26)
Self-employment	30 (52.63)	28 (32.56)
Farmer	3 (5.26)	9 (10.47)
Education		
No education	6 (10.53)	5 (5.81)
Primary	14 (24.56)	18 (20.93)
Secondary	28 (49.12)	62 (72.09)
A levels and above	9 (15.79)	1 (1.16)
Number of bereaved relatives, mean (SD)	2.33 (1.33)	2.05 (1.23)
Death was sudden vs gradual		
Sudden	45 (78.95)	72 (83.72)
Gradual	12 (21.05)	14 (16.28)
Death was expected vs unexpected		
Expected	5 (8.77)	12 (13.95)
Unexpected	52 (91.23)	74 (86.05)

^a^*n* = 2 missing

**Table 6:** Outcome scores for participants at baseline (*n* = 143), midline (*n* = 106) and endline (*n* = 98)

Outcomes	Intervention (*n* = 57)	Control (*n* = 86)
Shona Symptom Questionnaire total (0–14) (SSQ) at baseline (TO), mean (SD)^i^	10.37 (2.16)	8.83 (2.77)
Shona Symptom Questionnaire total (0–14) (SSQ) at midline (T1), mean (SD)^i^	9.48 (3.08)	8.59 (3.00)
Shona Symptom Questionnaire total (0–14) (SSQ) at endline (T2), mean (SD)^i^	9.48 (3.08)	8.57 (3.03)
Shona Symptom Questionnaire total (0–14) (SSQ) at baseline (TO), median (IQR)^i^	11 (9–12)	9 (7–11)
Shona Symptom Questionnaire total (0–14) (SSQ) at midline^i^ (T1), median (IQR)^i^	10 (7–11)	9.5 (8–10)
Shona Symptom Questionnaire total (0–14) (SSQ) at endlinei (T2), median (IQR)^i^	10 (7–11)	9.5 (7–10)
Medical outcome Study (MOS), Social Support Survey (SSS) total (18–90) at baseline (T0), mean (SD)^ii^	45.67 (16.02)^1^	51.85 (15.09)^2^
Medical outcome Study (MOS), Social Support Survey (SSS) total (18–90) at midline (T1), mean (SD)^ii^	50.28 (16.35)^3^	53.55 (16.02)^4^
Medical outcome Study (MOS), Social Support Survey (SSS) total (18–90) at endline (T2), mean (SD)^ii^	51.98 (17.89)^5^	54.57 (16.15)
Medical outcome Study (MOS), Social Support Survey (SSS) total (18–90) at baseline (T0), median (IQR)^ii^	43 (32–56)^2^	52 (39–66)^3^
Medical outcome Study (MOS), Social Support Survey (SSS) total (18–90) at midline (T1), median (IQR)^ii^	46 (37–60)^4^	53 (43–67)5
Medical outcome Study (MOS), Social Support Survey (SSS) total (18–90) at endline (T2), median (IQR)^ii^	52 (34–68)^6^	54 (43–69)
Texas Revised Inventory of Grief (TRIG) total scores (11–55), mean (SD) at baseline (T0)^iii^	17.24 (7.36)	19.53 (8.72)
Texas Revised Inventory of Grief (TRIG) total scores (11–55), mean (SD) at midline (T1)^iii^	17.85 (8.22)	20.09 (9.71)
Texas Revised Inventory of Grief (TRIG) total scores (11–55), mean (SD) at endline (T2)^iii^	18.27 (9.01)	19.22 (7.31)
Texas Revised Inventory of Grief (TRIG) total scores (11–55), median (IQR) at baseline (T0)^iii^	14 (11–22)	17 (12–24)
Texas Revised Inventory of Grief (TRIG) total scores (11–55), median (IQR) at midline (T1)^iii^	15.5 (11–20.5)	17.5 (12–25)
Texas Revised Inventory of Grief (TRIG) total scores (11–55), median (IQR) at endline (T2)^iii^	15 (11–23)	17.5 (15–21)

^1^*n* = 2 missing

^2﻿^*n* = 8 missing data

^3﻿^*n* = 11 missing

^4﻿^*n* = 3 missing

^5﻿^*n* = 1 missing

^6﻿^*n* = 7 missing

^i^SSQ (low scores better outcomes)

^ii^MOS-SSS (higher scores better outcomes)

^iii^TRIG (higher scores better outcomes)

**Table 7:** Change for outcome variables at baseline (*n* = 143), midline (*n* = 106) and endline (*n* = 98)

Shona Symptom Questionnaire (SSQ)	Intervention (*n* = 57)	Control (*n* = 86)
Shona Symptom Questionnaire total (SSQ) at baseline (T0), mean (SD)	10.37 (2.15)	8.83 (2.27)
Shona Symptom Questionnaire total (SSQ) at midline (T1), mean (SD)	9.48 (3.08)	8.59 (3.00)
Mean change (SD) from baseline	− .90 (3.89)	− .02 (3.83)
Shona Symptom Questionnaire total (SSQ) at end line (T2), mean (SD) (*n* = 52 missing)	9.48 (3.08)	8.57 (3.03)
Mean (SD) change from baseline	− .90 (3.89)	− .28 (3.79)
Mean (SD) change from midline	0	0
MOS-SSS		
Medical outcome Study (MOS), Social Support Survey (SSS) total at baseline (T0), mean (SD)	45.67 (16.02)^1^	51.85(15.09)^2^
Medical outcome Study (MOS), Social Support Survey (SSS) total at midline (T1), mean (SD)	50.28 (16.35)^3^	53.55 (16.02)^4^
Mean change (SD) from baseline	3.85 (23.19)	.9 (20.87)
Medical outcome Study (MOS), Social Support Survey (SSS) total at endline (T2), mean (SD)	51.5 (17.98)^5^	54.57 (16.15)
Mean change (SD) from baseline	7.92 (23.95)	2.52 (22.47)
Mean change (SD) from midline	2.73 (22.9)	1.2 (24.24)
Medical outcome Study (MOS), Social Support Survey (SSS) total (18–90) at baseline (T0), median (IQR)	43 (32–56)^6^	52 (39–66)^7^
Medical outcome Study (MOS), Social Support Survey (SSS) total (18–90) at midline (T1), median (IQR)	46 (37–60)^8^	53 (43–67)^9^
Median change from baseline	6 (− 13 to 20)	1 (− 14 to 14)
Medical outcome Study (MOS), Social Support Survey (SSS) total (18–90) at endline (T2), median (IQR)	52 (34–68)^10^	54 (43–69)
Median (IQR) change from baseline	8 (− 5–29)	6.5 (− 15–20.5)
Median (IQR) change from midline	2.5 (− 15–15)	3 (− 17–18)
Texas Revised Inventory (TRIG)		
TRIG mean (SD) at baseline (T0)	17.24 (7.36)	19.53 (8.72)
TRIG mean (SD) at midline (T1)	17.85 (8.22)	20.09 (9.71)
Mean change (SD) from baseline	.10 (9.64)	.65 (13.28)
TRIG mean (SD) at endline (T2)	18.27 (9.01)	19.22 (7.31)
Mean change (SD) from baseline	.52 (12.93)	.13 (10.36)
Mean change (SD) from midline	.42 (12.80)	− 1.34 (11.64)

^1^*n* = 8 missing data

^2^*n* = 11 missing

^3^*n* = 3 missing

^4^*n* = 1 missing

^5^*n* = 7 missing

^6^*n* = 8 missing

^7^*n* = 11 missing

^8^*n* = 3 missing

^9^*n* = 1 missing

^10^*n* = 7 missing

#### Baseline measures

Baseline measures for the Shona Symptom Questionnaire (SSQ) and the Texas Revised Inventory of Grief (TRIG) were broadly similar: the mean (SD) scores for the SSQ were 10.37 (2.16) in the intervention group compared with 8.83 (2.77) in the control group whilst the TRIG mean (SD) score at baseline were 17.24 (7.36) in the intervention group and 19.53 (8.72) in the control group. However, baseline scores for the Medical Outcome Study, Social Support Survey (MOS-SSS) were not similar. The mean (SD) scores in the intervention group were 45.67 (16.02) compared with 51.85 (15.09) in the control group.

#### Midline and endline measures (T1—3 months)

##### Retention of trial participants

Of the 143 participants randomized, 106 (52 intervention, 54 control) participants were followed up at 3 months (T1) and 98 were followed up at 6 months (T2) (52 intervention and 46 control).

A few challenges were experienced in setting the initial dates indicated for midline and endline data collection. These, however, did not affect the quality of the data collected. Challenges experienced were between midline and endline data collection periods, the dates had to be extended in line with post-election rules that forbade congregation in large numbers. These dates were further extended in response to a Cholera epidemic^1^ a few months after (see Table 8). Regulations in this time discouraged large gatherings as a way to curb infection. An additional challenge was that some of the participants’ contact details were either incorrect, unreachable or belonged to another person who would have had to convey new meeting dates on time if they could. Not all of them conveyed the messages in time.

**Table 8:** Data collection dates

Data collection	Community 1		Community 2	
	**Planned date**	**Actual date**	**Planned date**	**Actual date**
Baseline	6 March	6 March	7 March	7 March
Midline	6 June	12 July	7 June	13 July
Endline	6 September	16 November	7 September	30 October

Of the 98 participants who completed the trial, complete data were available for all the outcomes except the MOS-SSS which had *n* = 18 missing responses (*n* = 7 in the intervention group and *n* = 11 in the control group).

**Objective 5: Identification of trial participants’ views and experience of the intervention and its mechanisms of action**

Focus group discussions conducted with trial participants also indicated that a ripple effect was experienced with data showing that those individuals who the interventionists had initially reached out to, also reached out in turn to additional individuals suffering from loss within their own communities and families:

When we started this was just here in Chitungwiza but the program managed to help someone in Hurungwe, Mawere [rural area in Zimbabwe] (Female Respondent nine)

In the community those whom we talked to are now able to help others (Female Respondent Four)

There were several internal rewards that both the interventionists and the trial participants experienced and shared. Firstly, they reported that just by participating in a programme that allowed them to be open about the pain they experienced from the loss of loved ones was a process they appreciated. Secondly, it allowed them to heal and be able to share this process with those close to them. Thirdly, through their own capacity and self-motivation, they were able to share with those outside of their immediate communities.

Individually it helped me because I was one of the people who could not talk about these issues but now I can help others (Male Respondent Six)

The information we received on helping others who have lost their loved ones was really helpful to me, I sit down with them and explain to them…Some would ask if they will be able to get over it, but I encouraged them to accept it because they may become suicidal [if not supported] (Interventionist Ten)

**Objective 6: Estimating potential effect size**

Analysed data shows that the 9-cell bereavement tool was effective in that it allowed interventionists to share and learn from their own grief process. Participants were given a platform to openly talk about their grief in an open, safe space. Though they may have suffered loss from a long time before, because they had not processed the grief, they still acutely felt the pain.

It was very helpful for me to be able to deal with loss in my life… I have realized through the training and conversations with others that it is all part of life, we are now able to help others and to explain to them that this programme … has been helpful to me and how I can deal with my loss (Interventionist One)

The 9-cell bereavement tool allowed them to address this grief and pain. We do recognize that for some, more time to absorb the value of this new approach of understanding grief and expressing pain is required. This was also discerned when the intervention was delivered in Tamil Nadu, India[24] where it was found that group home visits following the exposure to the intervention helped facilitators recognize the value of the approach more deeply.

I faced loss recently, but I easily accepted it as a result of the training we received… I no longer think too much about my loss. Of course I can cry but it is better compared to before (Interventionist Six)

I am grateful for this programme because I have now accepted the death of my husband and I feel better. I was not able to work well but now I am stronger and can take care of the children (Interventionist Seven)

I lost a loved one in May and I was able to accept what had happened because of the training that we had received in March. It was easier for me to accept (Interventionist Two)

There were several internal rewards that both the interventionists and the trial participants experienced. Firstly, there was a benefit from participating in a programme that allowed them to be open about the pain they experienced from the loss of loved ones. Secondly, it allowed them to begin to accept the grief and bereavement process, and from there, be able to share this process with others. Thirdly, through their own capacity and self-motivation, they were able to share lessons they learnt about grief and the bereavement process with others.

After the training I was unfortunate to lose three very close relatives one after the other till about end of May. But I was quick to accept and move on and also counsel my other relatives (Interventionist One)

There has been some change because others will go for days without eating but now I have noticed change in those I have spoken to that they can eat normally even after the funeral (Interventionist Fourteen)

Data reinforces that participants reached out within their immediate vicinity, as was required in the study, but in addition, reached out beyond their own communities. A ripple effect was experienced with data showing that those who the interventionists had initially reached out to also reached out to additional individuals suffering from loss within their own communities and families.

Opportunities to support more people were attained and are ongoing with requests for wider exposure to the nine-cell bereavement tool, making sure to include people in the rural areas where exposure to such programmes was not as widespread or easily accessible.

There are some people who are far from here and also they need that help. There are some people who are lonely who don’t have someone to talk to. So, there is need that you help even those who will not be eating…The program is good because it helps heal the wounds you should also do it in the rural areas (Male Respondent Eleven)

If we are many we can also refer others to areas where some of us can be found (Male Respondent Five)

Media, including the radio, was suggested as an additional means of information distribution for a wider reach to rural areas.

These workshops will help them realize that they are not the only ones with problems…hence they will heal fast (Female Respondent Four)

I think it will be of much help if you put it on radio since this will help a lot of people like other programs on radio (Male Respondent One)

We can console others through the phone (Female Respondent Eight)

Requests for more information related to non-communicable diseases such as cancer, high blood pressure, stroke and diabetes were requested as community members felt that these ‘modern-day diseases’ were not well understood in their communities.

Collectively, these data establish the important role that a healthy grieving process can play in the lives of the bereaved; how context-specific, culturally appropriate and individually tailored support is important in light of the intersectionality of individual life experiences; and together highlight the need to conduct a rigorous evaluation of the nine-cell bereavement tool.

See Table 9 which summarizes all the above against feasibility criteria and questions.

**Table 9:** Summary of feasibility and success criteria

Questions and criteria	Findings
1. Will the processes of a randomized cluster trial be possible?	The processes of the randomized cluster trial were possible
2. Will we be able to recruit at least 75% of the suggested sample size within 3 weeks?	The study was able to recruit the suggested sample size within 3 weeks. Following the suggested trial recruitment criteria as indicated in the protocol, we managed to communicate and sensitize community leaders within one week; interventionists were recruited within one week and the trial participants were successfully recruited within another week The intended minimum numbers to be recruited were: • Interventionists: 50 in total • Trial participants: 100 in total The actual numbers recruited were: • Interventionists: 56, which is 12% more than the minimum suggested • Trial Participants: 135, which is 35% more than the minimum suggested
3. Will we be able to retain at least 75% of the trial participants in the 9 months of the study?	The study was able to retain at least 75% of the trial participants in the 9 months of the study To allow for any drop outs due to unforeseen challenges, we had recruited 25% above the minimum number we were expecting for the study. In addition and to support retention of participants, we recorded participants’ full names and contact details, which we then used remind participants of the data collection dates and venues as needed The minimum number of trial participants required for the study was 100 • This meant that we had to retain at least 75% (*n* = 75) • Results show that we recruited *n* = 135 and retained *n* = 108 at midline and *n* = 98 at endline Because we recruited higher than the minimum of 100, we managed to retain the minimum retention figure. In addition, we managed to retain 80% of total trial participants recruited
4. Can we deliver the 9-cell bereavement intervention?	The 9-cell bereavement intervention was delivered successfully
5. Is the intervention delivered as intended and does process data suggest the planned effect is likely?	The intervention was delivered as intended with analysed data showing the positive effect of the intervention: • Analysed data shows that the 9-cell bereavement tool was effective in that it allowed interventionists to share and learn from their own grieving process • Participants were given a platform to openly talk about their grief, in an open, safe space. Though they may have suffered loss in a time period that seemed a long time ago, because they had not processed the grief, they still felt the pain. Being taken through the 9-cell bereavement tool, allowed them to address this grief and pain • Data shows that participants reached out within their immediate vicinity, as required in the study, and in addition, reached out beyond their own communities • A ripple effect was experienced with data showing that those who the interventionists had initially reached out to also reached out to additional individuals suffering from loss within their own communities and families • There were several internal rewards that both the interventionists and the trial participants experienced. Firstly, just by participating in a program that allowed them to be open about the pain they experienced from the loss of loved ones. Secondly, it allowed them to heal and be able to share this process with others. Thirdly, through their own capacity and self-motivation, they were able to share lessons they learnt about grief and the bereavement process with others • Opportunities to support more people were attained and are ongoing with requests for wider exposure to the 9-cell bereavement tool, making sure to include people in the rural areas where exposure to such programs were not as widespread or easily accessible • Media, including the radio, was suggested as an additional means of information distribution for wider reach to rural areas • Requests and the need for more information related to non-communicable diseases such as cancer, high blood pressure, stroke and diabetes, was requested as community members felt that these ‘modern day diseases’ were not well understood in the communities

**Objective 7: Determining whether a full trial is warranted**

Given the success of implementing this randomized cluster trial in terms of recruitment and retention, successfully implementing the nine-cell bereavement tool; acceptability and completeness of measures and data; collection and documentation of trial participants’ views; positive experience of the intervention and its mechanisms of action; and, ability to estimate potential effect size; a full trial is warranted. In addition, a full trial will at the first level lend to the paucity in data on bereavement in Africa, and at the second level, provide data that both supports and promotes the need for and the success of locally based interventions in palliative care.

## Discussion

### The intervention and trial findings

We were able to both implement and evaluate the nine-cell bereavement tool at the community level and to evaluate it using a cluster randomized control trial design.

The processes of the randomized cluster trial were possible. The study was able to recruit the suggested sample size within 3 weeks. The study was able to retain at least 75% of the trial participants in the 9 months of the study. The nine-cell bereavement intervention was delivered as intended with analysed data showing the positive effect of the intervention.

Analysed data shows that the nine-cell bereavement tool was effective in that it allowed interventionists to share and learn from their own grieving process. Participants were given a platform to openly talk about their grief, in an open, safe space. Though they may have suffered loss a long time in the past (for example more than 18 months ago) because they had not processed the grief, they still felt the pain. Being taken through the nine-cell bereavement tool, allowed them to address this grief and pain.

Data shows that participants reached out within their immediate vicinity, as was required in the study, but managed to reach even further. The ripple effect of much-needed internal rewards was experienced. The need and therefore opportunity to support even more people were shared. To reach more people and more widely, the use of different communication mediums was encouraged.

### Trial limitations

As the focus of the study was resource-led and so laser-focused on the aim of assessing the feasibility of implementing the nine cell bereavement intervention, we did not collect qualitative data from the trial participants on the experience they had from the interventionists who delivered the intervention in the communities. We do, however, have anecdotal data from the interventionists themselves on the effectiveness of the tool, from their own feedback in communication with the trial participants. The evidence indicated that trial participants positively experienced the intervention, allowing them to share it with loved ones both in their community and in their rural areas, who had been bereaved. Interviews with trial participants can be formalized for future studies.

There were a few instances where interventionists, that is, those who were taken through the intervention by our facilitators, revealed in their contributions during the intervention, that they may have needed more time to be immersed into the concept of the nine-cell tool. For future interventions, it is to be noted that though the nine-cell process is structured as a full-day session, the number of days is flexible to allow for deeper immersion around different aspects that each community and group may need depending on their understanding and contextual relationships with death.

We were unable to calculate confidence intervals because our study estimated the recruitment and retention rates for interventionists *n* = 50 and trial participants *n* = 100. These figures were based on recommended sample sizes from past feasibility studies. The past feasibility studies cited are listed in the ‘Recruitment of trial participants’ section in this paper. We accept this as a limitation.

Due to communication processes and resources, it was not possible for this study to blind the researchers on which was the control group and which was the intervention group. However, neither of the communities was aware. Contamination questions used also showed that there was no interaction of the participants from each group throughout the duration of the study.

With regard to differential attrition, there was a higher loss to follow-up of trial participants, in the control group. This higher loss to follow-up of trial participants was because of communication challenges within a smaller time frame compared to the time frame provided for the intervention group.

These challenges can be reduced in future studies, by being more proactive with communication, and within longer time frames so that participants are alerted and prepared well in advance. Some of the ways this can be done is by having a participant locator form that has alternate ways of reaching participants for reminders and for any change of dates of data collection.

In addition, alternate ways of reaching participants would include contact details of next of kin and or other persons in their vicinity who would be able to better reach them, should their primary contact details not be available at the time of contact. The longer time frame will accommodate other ways of contacting participants should the first point of contact is not successful.

### Generalizability

The scales used for this study, that is, the SSQ, the MOSSS and the TRIG, were effective in that the SSQ was locally developed and has been successfully tested in other countries in the region and in another low-income country, India [24]. It was therefore locally, culturally and contextually appropriate for Zimbabwe. The MOSSS and the TRIG have been tested for validity in different low to middle-income spaces around the world, allowing them to be feasible for the Zimbabwean context. The combination of both local, contextual and inclusive scales is encouraged in all studies for relevance, feasibility and validity. As a result, effective delivery processes and resulting uptake are increased.

### Strengths of the study

This was the first time this locally and contextually developed bereavement tool was tested and successfully implemented in Zimbabwe. The need for rigorous evaluation of scarce, culturally specific and community-based interventions in sub-Saharan Africa highlights the urgent need to carry out a full trial in this subject matter.

The study was successfully carried out by local researchers with support from the communities, and locally based institutions and organizations already conducting work in the communities. Support and direction throughout the trial were further supported by Kings College London researchers. This collaborative nature allowed for richness in both development and delivery with easier uptake because of the urgency and need of bereavement studies and interventions.

## Conclusion

Our literature search and protocol indicated that bereavement is an under-researched field and that bereavement interventions are rarely described within African palliative care intervention studies. Community-based interventions using already existing structures especially in low-resources countries are more sustainable. In addition, there is an increase in reach especially when locally available lay health workers are part of the interventions.

Research continues to show that bereavement and bereavement interventions are an essential and core component of palliative care, and more so in low-resourced countries whose health systems are already struggling from high mortality rates in HIV, tuberculosis, cancer, malaria, suicide and other communicable diseases. A full trial is not only warranted as a contribution to the currently sparse literature, but it will have an enormous potential public health benefit in supporting and saving lives in many more under-resourced and under-supported countries. The next logical step toward a full trial that would be an internal pilot that will take into account the lessons learnt through the limitations of this study, and from there, the development of a full trial.

## Supplementary Information

**Additional file 1.** Flow diagram.

**Additional file 2: Table 1.** Sample size and recruitment rates of interventionists. **Table 2.** Sample size and recruitment rates trial participants. **Table 3.** Retention of interventionists. **Table 4.** Retention of trial participants. **Table 5.** Demographics of the comparison of the participants from the two communities. **Table 6.** Outcomes scores for participants at baseline (*n* = 143), midline (*n* = 106) and endline (*n* = 98). **Table 7.** Change for outcome variables at baseline (*n* = 143), midline (*n* = 106) and endline (*n* = 98). **Table 8.** Data collection dates. **Table 9.** Summary results against feasibility questions. **Figure 1.** Nine cell bereavement tool. **Figure 2.** Flow Diagram (placed in a separate document).

## Data Availability

Datasets generated during and analysed during the current study are not publicly available in regard to the confidentiality of participants but are available from the corresponding author upon reasonable request.

## Footnotes

1 WHO Emergences preparedness response: Cholera_Zimbabwe: https://www.who.int/csr/don/05-october-2018-cholera-zimbabwe/en/
