# Supplementary material for: Improving bereavement outcomes in Zimbabwe: results of a feasibility cluster trial of the 9-cell bereavement tool
Source: Pilot Feasibility Stud. 2023 Jul 21;9:127. doi: 10.1186/s40814-023-01313-2 (PMC10360285; doi:10.1186/s40814-023-01313-2)
Supplement: Supplementary file 1 — Additional file 1. Flow diagram. [file 40814_2023_1313_MOESM1_ESM.docx]

Baseline assessments (n= (150-7)= 143)

Recruitment rate: 143/150 = 95

Excluded (n=7)

- Reason for exclusion: They did not have an invitation letter or name of referring interventionists

Follow-up at midline at 3 months (T1) (n=52)

- Retention rate between baseline and midline 52/57= 91%

**Lost to follow-up** (n=5 at midline) Reasons: cholera outbreak; national elections and incorrect or unreachable contact numbers disrupted dates of data collection and the ability to communicate to all participants

**Discontinued intervention** (n=0)

Allocated to intervention (n=57 Intervention group)

- Received allocated intervention after baseline data collection(n=57)
- Did not receive allocated intervention (n=0)

**Lost to follow-up** (n=32 at midline) Reasons: Cholera outbreak; National elections; Incorrect or unreachable contact numbers.

**Discontinued intervention** (n=0)

Allocated to intervention (n=86 Control group)

- Received allocated intervention after endline data collection(n=86)
- Did not receive allocated intervention (n=0)

Follow-up at end line (T2) (n=46)

- Retention rate between midline and endline 46/54= 85%
- Retention rate between baseline and endline 46/86= 53%

## Allocation

## Assessment

## Follow-Up

Randomized (n=143)

These are Trial participants from 2 communities

## Enrollment

Screened for eligibility assessment (n=150)

## Screened

**Loss to follow-up** (n=8) at endline). Reasons: Incorrect or unreachable contact numbers

**Discontinued intervention** (n=0)

Follow-up at midline at 3 months (T1) (n=54)

- Retention rate between baseline and midline 54/86= 62%

Follow-up at endline (T2) (n=52)

- Retention rate between midline and endline 100%
- Retention rate between baseline & endline = 91%
