# Supplementary material for: Improving bereavement outcomes in Zimbabwe: results of a feasibility cluster trial of the 9-cell bereavement tool
Source: Pilot Feasibility Stud. 2023 Jul 21;9:127. doi: 10.1186/s40814-023-01313-2 (PMC10360285; doi:10.1186/s40814-023-01313-2)
Supplement: Supplementary file 2 — Additional file 2: Table 1. Sample size and recruitment rates of interventionists. Table 2. Sample size and recruitment rates trial participants. Table 3. Retention of interventionists. Table 4. Retention of trial participants. Table 5. Demographics of the comparison of the participants from the two communities. Table 6. Outcomes scores for participants at baseline (n = 143), midline (n = 106) and endline (n = 98). Table 7. Change for outcome variables at baseline (n = 143), midline (n = 106) and endline (n = 98). Table 8. Data collection dates. Table 9. Summary results against feasibility questions. Figure 1. Nine cell bereavement tool. Figure 2. Flow Diagram (placed in a separate document). [file 40814_2023_1313_MOESM2_ESM.docx]

**TITLE**

**IMPROVING BEREAVEMENT OUTCOMES IN ZIMBABWE: RESULTS OF A FEASIBILITY CLUSTER TRIAL OF THE 9-CELL BEREAVEMENT TOOL**

**TABLES & FIGURES**

**TABLES**

Table 1. Sample size and recruitment rates of interventionists.

Table 2. Sample size and recruitment rates trial participants.

Table 3. Retention of interventionists

Table 4. Retention of trial participants

Table 5. Demographics of the comparison of the participants from the two communities

Table 6. Outcomes scores for participants at baseline (n=143), midline (n=106) and endline (n=98).

Table 7. Change for outcome variables at baseline (n=143), midline (n=106) and endline (n=98).

Table 8. Data collection dates.

Table 9. Summary results against feasibility questions.

**FIGURES**

1. Figure 1: Nine cell bereavement tool
2. Figure 2: Flow Diagram (placed in a separate document)

**Table 1: Interventionists recruitment rates**

| Interventionists | Target recruitment (n) | Actual recruited (n) | Percentage (%) of target actually recruited |
| --- | --- | --- | --- |
| Community 1 | 25 | 25 | 25 = 100% |
| Community 2 | 25 | 31 | 31 = 112% |
| Total | 50 | 56 | 56 = 112% |

**Table 2: Trial participants recruitment rates**

| Trial participants | Target recruitment (n) | Actual recruited (n) | Percentage (%) of target actually recruited |
| --- | --- | --- | --- |
| Community 1 | 50 – 75 | 57 | 57 = 114% |
| Community 2 | 50 – 75 | 86 | 86 = 172% |
| Total | 100 – 150 | 143 | 143 = 143% |

**Table 3: Retention rates of Interventionists**

|  | Actual recruited (n) | Actual retained at midline (n) | Actual retained at endline (n) | Actual (%) retained |
| --- | --- | --- | --- | --- |
| Community 1 Interventionists | 25 | 25 | 25 | 100+% |
| Community 2 Interventionists | 31 | 31 | 31 | 100+% |
| **Total** | **56** | **56** | **56** | **100%** |

**Table 4: Retention rates of trial participants**

|  | Actual recruited | Actual retained at midline | Actual retained at endline | Actual (%) retained |
| --- | --- | --- | --- | --- |
| Community 1  Trial Participants | 57 | 52 | 52 | 52/57 = 91% base to midline  52/52 = 100% mid to endline  52/57 = 91% base to endline |
| Community 2  Trial Participants | 86 | 54 | 46 | 54/86 = 62% base to midline  46/54 = 85% mid to endline  46/86 = 53% base to endline |
| **Total** | **143** | **106** | **98** | **106/143 = 74% base to midline**  **98/106 = 92% mid to endline**  **98/143 = 69% base to endline** |

**Table 5: Demographics of the comparison of the participants from the two communities**

| Variables | Intervention (Seke) (n=57) | Control (St Mary's) (n=86) |
| --- | --- | --- |
| Age in years^[[1]](#footnote-1)^  18-25  26-35  36-45  46-55  46/max | 3 (5.26)  11 (19.30)  16 (28.07)  13 (22.81)  14 (24.56) | 17 (20)  19 (22.35)  25 (29.41)  14 (16.47)  10 (11.76) |
| Male/Female gender | 11 (19.30)/ 46 (80.70) | 13 (15.12)/ 73 (84.88) |
| Black race/other race | 56 (98.25)/1 (1.75) | 85 (98.84)1 (1.16) |
| Importance of religious beliefs |  |  |
| Not important at all | 1 (1.75) | 0 |
| Not very important | 1 (1.75) | 1 (1.16) |
| Fairly important | 2 (3.51) | 1 (1.16) |
| Very important | 53 (92.98) | 83 (96.51) |
| Don’t know | 0 | 1 (1.16) |
| Religion |  |  |
| Catholic | 7 (12.28) | 9 (10.47) |
| Presbyterian | 15 (26.32) | 17 (19.77) |
| Apostolic | 10 (17.54) | 19 (22.09) |
| Pentecostal | 18 (31.58) | 26 (30.23) |
| Other | 7 (12.28) | 15 (17.44) |
| Employment |  |  |
| Not employed | 18 (31.58) | 29 (33.72) |
| Formally Employed | 6 (10.53) | 20 (23.26) |
| Self-employment | 30 (52.63) | 28 (32.56) |
| Farmer | 3 (5.26) | 9 (10.47) |
| Education |  |  |
| No education | 6 (10.53) | 5 (5.81) |
| Primary | 14 (24.56) | 18 (20.93) |
| Secondary | 28 (49.12) | 62 (72.09) |
| A levels and above | 9 (15.79) | 1 (1.16) |
| Number of bereaved relatives: mean (SD) | 2.33 (1.33) | 2.05 (1.23) |
| Death was Sudden vs gradual |  |  |
| Sudden  Gradual | 45 (78.95)  12 (21.05) | 72 (83.72)  14 (16.28) |
| Death was expected vs unexpected |  |  |
| Expected  Unexpected | 5 (8.77)  52 (91.23) | 12 (13.95)  74 (86.05) |

**Table 6: Outcome scores for participants at baseline (n=143) , midline (n=106) and endline (n=98)**

| Outcomes | Intervention (n=57) | Control n=86 |
| --- | --- | --- |
| Shona Symptom Questionnaire total (0-14) (SSQ) at baseline (TO) mean (SD)^i^ | 10.37 (2.16) | 8.83 (2.77) |
| Shona Symptom Questionnaire total (0-14) (SSQ) at midline (T1) mean (SD)^i^ | 9.48 (3.08) | 8.59 (3.00) |
| Shona Symptom Questionnaire total (0-14) (SSQ) at endline (T2) mean (SD)^i^ | 9.48 (3.08) | 8.57 (3.03) |
| Shona Symptom Questionnaire total (0-14) (SSQ) at baseline (TO) median (IQR)^i^ | 11 (9-12) | 9 (7-11) |
| Shona Symptom Questionnaire total (0-14) (SSQ) at midlineI (T1) median (IQR)^i^ | 10 (7-11) | 9.5 (8-10) |
| Shona Symptom Questionnaire total (0-14) (SSQ) at endlinei (T2) median (IQR)^i^ | 10 (7-11) | 9.5 (7-10) |
| Medical outcome Study (MOS), Social Support Survey (SSS) total (18-90) at baseline (T0) mean (SD)^ii^ | 45.67 (16.02)^[[2]](#footnote-2)^ | 51.85(15.09)^[[3]](#footnote-3)^ |
| Medical outcome Study (MOS), Social Support Survey (SSS) total (18-90) at midline (T1) mean (SD)^ii^ | 50.28 (16.35)^[[4]](#footnote-4)^ | 53.55 (16.02)^[[5]](#footnote-5)^ |
| Medical outcome Study (MOS), Social Support Survey (SSS) total (18-90) at endline (T2) mean (SD)^ii^ | 51.98 (17.89)^[[6]](#footnote-6)^ | 54.57 (16.15) |
| Medical outcome Study (MOS), Social Support Survey (SSS) total (18-90) at baseline (T0) median (IQR)^ii^ | 43 (32-56)3 | 52 (39-66)4 |
| Medical outcome Study (MOS), Social Support Survey (SSS) total (18-90) at midline (T1) median (IQR)^ii^ | 46 (37-60)5 | 53 (43-67)6 |
| Medical outcome Study (MOS), Social Support Survey (SSS) total (18-90) at endline (T2) median (IQR)^ii^ | 52 (34-68)7 | 54 (43-69) |
| Texas Revised Inventory of Grief (TRIG) total scores (11-55) mean (SD) at baseline (T0)^iii^ | 17.24 (7.36) | 19.53 (8.72) |
| Texas Revised Inventory of Grief (TRIG) total scores (11-55) mean (SD) at midline (T1)^iii^ | 17.85 (8.22) | 20.09 (9.71) |
| Texas Revised Inventory of Grief (TRIG) total scores (11-55) mean (SD) at endline (T2)^iii^ | 18.27 (9.01) | 19.22 (7.31) |
| Texas Revised Inventory of Grief (TRIG) total scores (11-55) median (IQR) at baseline (T0)^iii^ | 14 (11-22) | 17 (12-24) |
| Texas Revised Inventory of Grief (TRIG) total scores (11-55) median (IQR) at midline (T1)^iii^ | 15.5 (11-20.5) | 17.5 (12-25) |
| Texas Revised Inventory of Grief (TRIG) total scores (11-55) median (IQR) at endline (T2)^iii^ | 15 (11-23) | 17.5 (15-21) |

i SSQ (low scores better outcomes)

^ii^ MOS-SSS (higher scores better outcomes)

^iii^ TRIG (higher scores better outcomes)

**Table 7: Change for outcome variables at baseline (n=143), midline (n=106) and endline (n=98)**

| Shona Symptom Questionnaire (SSQ) | Intervention (n=57) | Control (n=86) |
| --- | --- | --- |
| Shona Symptom Questionnaire total (SSQ) at baseline (T0) mean (SD) | 10.37 (2.15) | 8.83 (2.27) |
| Shona Symptom Questionnaire total (SSQ) at midline (T1) mean (SD) | 9.48 (3.08) | 8.59 (3.00) |
| Mean change (SD) from baseline | -.90 (3.89) | -.02 (3.83) |
| Shona Symptom Questionnaire total (SSQ) at end line (T2) mean (SD) (n=52 missing) | 9.48 (3.08) | 8.57 (3.03) |
| Mean (SD) change from baseline | -.90 (3.89) | -.28 (3.79) |
| Mean (SD) change from midline | 0 | 0 |
| MOS-SSS |  |  |
| Medical outcome Study (MOS), Social Support Survey (SSS) total at baseline (T0) mean (SD) | 45.67 (16.02)^[[7]](#footnote-7)^ | 51.85(15.09)^[[8]](#footnote-8)^ |
| Medical outcome Study (MOS), Social Support Survey (SSS) total at midline (T1) mean (SD) | 50.28 (16.35)^[[9]](#footnote-9)^ | 53.55 (16.02)^[[10]](#footnote-10)^ |
| Mean change (SD) from baseline | 3.85 (23.19) | .9 (20.87) |
| Medical outcome Study (MOS), Social Support Survey (SSS) total at endline (T2) mean (SD) | 51.5 (17.98)^[[11]](#footnote-11)^ | 54.57 (16.15) |
| Mean change (SD) from baseline | 7.92 (23.95) | 2.52 (22.47) |
| Mean change (SD) from midline | 2.73 (22.9) | 1.2 (24.24) |
| Medical outcome Study (MOS), Social Support Survey (SSS) total (18-90) at baselineii (T0) median (IQR) | 43 (32-56)^[[12]](#footnote-12)^ | 52 (39-66)^[[13]](#footnote-13)^ |
| Medical outcome Study (MOS), Social Support Survey (SSS) total (18-90) at midlineii (T1) median (IQR) | 46 (37-60)^[[14]](#footnote-14)^ | 53 (43-67)^[[15]](#footnote-15)^ |
| median change from baseline | 6 (-13 to 20) | 1 (-14 to 14) |
| Medical outcome Study (MOS), Social Support Survey (SSS) total (18-90) at endlineii (T2) median (IQR) | 52 (34-68)^[[16]](#footnote-16)^ | 54 (43-69) |
| median (IQR) change from baseline | 8 (-5 to 29) | 6.5 (-15 to 20.5) |
| median (IQR) change from midline | 2.5 (-15 to 15) | 3 (-17 to 18) |

| Texas Revised Inventory (TRIG) |  |  |
| --- | --- | --- |
| TRIG mean (SD) at baseline (T0) | 17.24 (7.36) | 19.53 (8.72) |
| TRIG mean (SD) at midline (T1) | 17.85 (8.22) | 20.09 (9.71) |
| Mean change (SD) from baseline | .10 (9.64) | .65 (13.28) |
| TRIG mean (SD) at endline (T2) | 18.27 (9.01) | 19.22 (7.31) |
| Mean change (SD) from baseline | .52 (12.93) | .13 (10.36) |
| Mean change (SD) from midline | .42 (12.80) | -1.34 (11.64) |

**Table 8: Data collection dates**

| **Data Collection** | **Seke** | | | **St. Mary’s** | |
| --- | --- | --- | --- | --- | --- |
|  | **Planned date** | **Actual date** | **Planned date** | | **Actual date** |
| Baseline | 6 March | 6 March | 7 March | | 7 March |
| Midline | 6 June | 12 July | 7 June | | 13 July |
| Endline | 6 September | 16 November | 7 September | | 30 October |

**Table 9: Summary of feasibility and success criteria**

| Questions and Criteria | Findings |
| --- | --- |
| 1. Will the processes of a randomized cluster trial be possible? | The processes of the randomized cluster trial were possible. |
| 1. Will we be able to recruit at least 75% of the suggested sample size within 3 weeks? | The study was able to recruit the suggested sample size within 3 weeks. Following the suggested trial recruitment criteria as indicated in the protocol, we managed to communicate and sensitize community leaders within one week; interventionists were recruited within one week and the trial participants were successfully recruited within another week.  The intended minimum numbers to be recruited were:   - Interventionists: 50 in total - Trial participants: 100 in total   The actual numbers recruited were:   - Interventionists: 56, which is 12% more than the minimum suggested - Trial Participants: 135, which is 35% more than the minimum suggested |
| 1. Will we be able to retain at least 75% of the trial participants in the 9 months of the study? | The study was able to retain at least 75% of the trial participants in the 9 months of the study.  To allow for any drop outs due to unforeseen challenges, we had recruited 25% above the minimum number we were expecting for the study. In addition and to support retention of participants, we recorded participants’ full names and contact details, which we then used remind participants of the data collection dates and venues as needed.  The minimum number of trial participants required for the study was 100   - This meant that we had to retain at least 75% (n=75) - Results show that we recruited n=135 and retained n=108 at midline and n=98 at endline.   Because we recruited higher than the minimum of 100, we managed to retain the minimum retention figure. In addition, we managed to retain 80% of total trial participants recruited. |
| 1. Can we deliver the 9-cell bereavement intervention? | The 9-cell bereavement intervention was delivered successfully. |
| 1. Is the intervention delivered as intended and does process data suggest the planned effect is likely? | The intervention was delivered as intended with analysed data showing the positive effect of the intervention:   - Analysed data shows that the 9-cell bereavement tool was effective in that it allowed interventionists to share and learn from their own grieving process. - Participants were given a platform to openly talk about their grief, in an open, safe space. Though they may have suffered loss in a time period that seemed a long time ago, because they had not processed the grief, they still felt the pain. Being taken through the 9-cell bereavement tool, allowed them to address this grief and pain. - Data shows that participants reached out within their immediate vicinity, as required in the study, and in addition, reached out beyond their own communities. - A ripple effect was experienced with data showing that those who the interventionists had initially reached out to also reached out to additional individuals suffering from loss within their own communities and families. - There were several internal rewards that both the interventionists and the trial participants experienced. Firstly, just by participating in a program that allowed them to be open about the pain they experienced from the loss of loved ones. Secondly, it allowed them to heal and be able to share this process with others. Thirdly, through their own capacity and self-motivation, they were able to share lessons they learnt about grief and the bereavement process with others. - Opportunities to support more people were attained and are ongoing with requests for wider exposure to the 9-cell bereavement tool, making sure to include people in the rural areas where exposure to such programs were not as widespread or easily accessible - Media, including the radio, was suggested as an additional means of information distribution for wider reach to rural areas - Requests and the need for more information related to non-communicable diseases such as cancer, high blood pressure, stroke and diabetes, was requested as community members felt that these ‘modern day diseases’ were not well understood in the communities. |

**FIGURES**

**Figure 1: Nine cell bereavement tool**


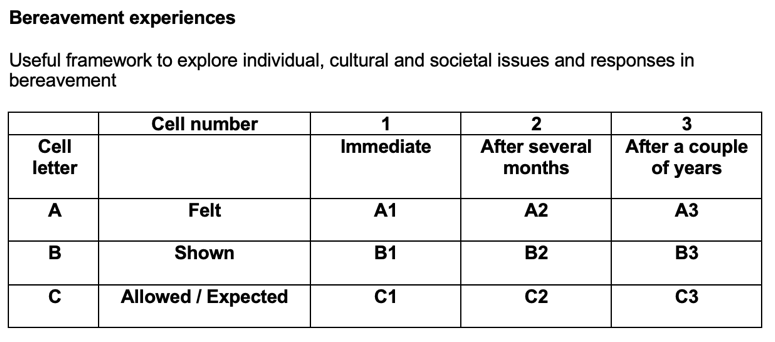


**Figure 2: CONSORT flow diagram (in a separately attached document)**

1. n=2 missing [↑](#footnote-ref-1)
2. n=8 missing data [↑](#footnote-ref-2)
3. n=11 missing [↑](#footnote-ref-3)
4. n=3 missing [↑](#footnote-ref-4)
5. n=1 missing [↑](#footnote-ref-5)
6. n=7 missing [↑](#footnote-ref-6)
7. n=8 missing data [↑](#footnote-ref-7)
8. n=11 missing [↑](#footnote-ref-8)
9. n=3 missing [↑](#footnote-ref-9)
10. n=1 missing [↑](#footnote-ref-10)
11. n=7 missing [↑](#footnote-ref-11)
12. n=8 missing [↑](#footnote-ref-12)
13. n=11 missing [↑](#footnote-ref-13)
14. n=3 missing [↑](#footnote-ref-14)
15. n=1 missing [↑](#footnote-ref-15)
16. n=7 missing [↑](#footnote-ref-16)
